# Surfactant Compatibility Screening Improves Formulation Performance of *Metarhizium anisopliae* for Control of the Tomato Leafminer *Phthorimaea absoluta*

**DOI:** 10.3390/insects17101014

**Published:** 2026-10-01

**Authors:** Song Mei, Amr Abou El-Ela, Yuanqiu Dong, Asim Munawar, Shuzhen Li, Xiaolong Liu, Wenwu Zhou, Yaqiang Zheng

**Affiliations:** 1 College of Pharmacy, Guizhou University of Traditional Chinese Medicine, Guiyang 550025, China; 2 Ministry of Agriculture and Rural Affairs Key Laboratory of Molecular Biology of Crop Pathogens and Insects & Key Laboratory of Biology of Crop Pathogens and Insects of Zhejiang Province, Institute of Insect Sciences, Zhejiang University, Hangzhou 310058, China; 3 Plant Protection Department, Faculty of Agriculture (Saba Basha), Alexandria University, Alexandria 21531, Egypt

**Keywords:** abiotic stress tolerance, conidial dispersibility, entomopathogenic fungi, fungal biopesticides, *Metarhizium anisopliae*, *Phthorimaea absoluta* (Meyrick), surfactant compatibility

## Abstract

The tomato leafminer, *Phthorimaea absoluta* (Meyrick), is one of the most destructive pests of tomato worldwide, and its management is increasingly constrained by insecticide resistance and the pest’s concealed feeding behavior. Entomopathogenic fungi such as *Metarhizium anisopliae* represent promising biological alternatives; however, the hydrophobic nature of fungal conidia often limits their dispersibility and field performance. In this study, the compatibility of *M. anisopliae* strain QLSMA201102 with 21 commercial surfactants was systematically evaluated to identify formulation additives that improve conidial performance without compromising fungal viability. Surfactants representing four classes (non-ionic, anionic, biosurfactant, and silicone-based) were screened for their effects on conidial germination, mycelial growth, foliar adherence, dispersibility, environmental stress tolerance, and insecticidal activity against *P. absoluta*. Several anionic surfactants strongly inhibited conidial germination, whereas PEG-8000, Tween-80, and the biosurfactant SL maintained high viability and significantly improved conidial dispersibility and foliar retention. PEG-8000 provided the strongest protection against UV radiation (>90% germination after UV-A exposure), while SL enhanced the thermotolerance of conidia. In larval bioassays, formulations containing SL, PEG-8000 and Tween-80 significantly increased mortality of *P. absoluta* to 91.49%, 91.66% and 91.57%, respectively, compared to 65.71% for the surfactant-free control conidia. Overall, these results identify PEG-8000, Tween-80, and SL as promising formulation additives for improving the stability and efficacy of *M. anisopliae*-based biopesticides. This work provides a foundation for developing optimized fungal formulations to support sustainable integrated pest management of *P. absoluta*.

## Simple Summary

The tomato leafminer, *Phthorimaea absoluta* (Meyrick), is a destructive invasive pest that feeds internally on tomato foliage and fruit, rendering chemical control both difficult and increasingly ineffective due to rising insecticide resistance. Entomopathogenic fungi such as *Metarhizium anisopliae* offer a sustainable alternative, yet their hydrophobic conidia tend to aggregate in aqueous suspensions, impairing dispersibility and field performance. Here, we screened 21 commercial surfactants from four chemical classes to identify additives that improve conidial suspension quality and stress tolerance without compromising fungal viability. Three candidates, the non-ionic surfactants PEG-8000 and Tween-80, and the biosurfactant sophorolipid (SL), significantly enhanced conidial dispersibility and foliar adhesion. Notably, PEG-8000 provided strong protection against UV-A radiation (>90% germination), while SL markedly improved thermotolerance. In bioassays, formulations combining these surfactants with *M. anisopliae* conidia achieved ~91% larval mortality against *P. absoluta*, compared with ~65% for conidia alone. These results show that careful surfactant selection markedly improves conidial delivery and efficacy. Our findings provide a practical basis for optimizing fungal biopesticide formulations.

## 1. Introduction

Entomopathogenic fungi (EPF), particularly those in the genera *Beauveria* and *Metarhizium*, are important biological control agents because they infect a broad range of arthropod pests via contact and can be incorporated into integrated pest management programs [1,2,3]. Among them, *Metarhizium anisopliae* (Hypocreales: Clavicipitaceae) has shown strong potential for suppressing major agricultural pests, including *Phthorimaea absoluta* (Meyrick) [4,5]. In addition to direct pathogenicity, some *Metarhizium* isolates can colonize plant tissues endophytically and may promote plant growth, induce host defenses, and suppress herbivores and phytopathogens [6,7]. Accordingly, the practical value of EPF depends not only on intrinsic virulence, but also on whether fungal propagules can be produced, formulated, stored, and delivered in a manner that preserves biological activity under field conditions [1].

A major obstacle to EPF field performance is the hydrophobic nature of their conidia [1,8]. This surface hydrophobicity originates largely from a rodlet layer composed of cell-wall-bound hydrophobin proteins together with surface lipids and wall-associated proteins, which reduces water wettability and promotes aggregation of conidia in aqueous suspensions [9,10]. Such aggregation reduces dispersibility, spray uniformity, and deposition on target surfaces [9,10]. In commercial mass production, conidia are usually dehydrated to establish metabolic dormancy and extend shelf-life [11,12,13]. However, these dehydrated propagules require suitable adjuvants upon rehydration to restore metabolic activity, achieve homogeneous dispersion, and guarantee effective deposition on target surfaces [14,15,16]. Surfactants represent one class of essential adjuvants: they facilitate harvesting hydrophobic conidia from solid-state culture, enable preparation of uniform suspensions for bioassays and field spraying, and serve as core components of suspension-concentrate biopesticide formulations [14,17]. Even so, surfactant application carries risks: compounds improving physical dispersion may concurrently damage fungal cel-membrane integrity, leading to reduced germination and infectivity, and these toxic effects are strongly dependent on chemical structure and dosage [15,16,18,19]. Balancing these benefits and risks requires deliberate formulation design. Formulation, therefore, plays a decisive role in determining whether promising fungal isolates can be translated into effective mycoinsecticides [1,20]. In particular, because EPFs are commonly applied as sprayable suspensions, formulation additives must not only stabilize and disperse conidia but also protect propagules from environmental stress and maintain their infectivity after application [1,20,21].

Surfactants are widely used in agrochemical formulations because they reduce surface tension and improve wetting, spreading, and dispersion [22,23]. In EPF systems, these properties may improve the quality of conidial suspension and promote contact with hydrophobic insect cuticles [20,24]. However, the formulation value of a surfactant cannot be judged solely by its physicochemical effects, because the same additive that improves suspension behavior may also impair conidial germination, viability, or virulence depending on its chemistry and concentration [25]. Accordingly, surfactant selection should be based on empirical compatibility screening rather than assumed equivalence among surfactant classes [25]. This is especially important in EPF-based biopesticides, where biological compatibility and application performance must be optimized simultaneously.

The tomato leafminer, *Phthorimaea absoluta* (Meyrick) = *Tuta absoluta* (Meyrick), is one of the most destructive invasive pests of tomato worldwide [26,27]. Since expanding beyond its native South American range, it has become widely established across Africa, Asia, and Europe, where larval feeding within leaves, stems, buds, and fruits causes substantial yield and quality losses [27,28]. Its concealed feeding habit, short generation time, and ability to persist under intensive production systems complicate management and strengthen the need for sustainable alternatives to heavy reliance on insecticides [21,29]. EPF-based biocontrol is increasingly recognized as a promising component of integrated pest management for *P. absoluta*, although formulation and delivery limitations still constrain broader adoption [21]. Despite this promise, relatively few studies have systematically evaluated how commercial surfactants influence multiple field-relevant traits of EPF propagules within a single screening framework for this pest pathosystem.

In previous work, we identified the native strain *M. anisopliae* QLSMA201102 as a promising candidate for the biological control of *P. absoluta* in tomato [5,30]. However, its practical deployment requires formulation components that improve conidial handling and application performance without compromising fungal viability or insecticidal activity. Here, we screened 21 commercial surfactants representing four categories (nonionic, anionic, biosurfactant, and silicone-based) for their effects on key performance traits of *M. anisopliae* conidia, including viability, dispersibility, stress tolerance, and insecticidal efficacy. By integrating physicochemical and biological compatibility traits into a comparative evaluation framework, this study seeks to identify surfactants that are genuinely suitable for formulation development and to provide a more rational basis for improving EPF-based biopesticides for sustainable management of *P. absoluta* in tomato production systems.

## 2. Materials and Methods

### 2.1. Plant and Insect Cultures

Tomato seeds of the cultivar Shouxin were purchased from Shouguang Xinxinran Horticulture Co., Ltd. (Shouguang, China). Seeds were surface-sterilized in 1% sodium hypochlorite (NaClO) for 5 min, rinsed three times with sterile distilled water, and dried on sterile filter paper. The sterilized seeds were then sown individually in 4 × 4 × 4 cm pots containing nutrient soil obtained from the same supplier. Tomato plants approximately 30 cm in height were used for maintenance of the insect colony.

Colonies of *P. absoluta* were established from field-collected larvae obtained from naturally infested tomato plants in agricultural areas near Zhijin City, Guizhou Province, China (26°40′60″ N, 106°39′90″ E). Laboratory colonies were maintained on potted tomato plants in custom-designed plexiglass rearing cages (40 × 40 × 40 cm; length × width × height) with two opposite sides covered by 200-mesh nylon screen for ventilation. Adult moths were provided continuously with freshly prepared 5% (*v*/*v*) honey solution using cotton-wick feeders. To obtain synchronized insect cohorts for bioassays, gravid females were confined with fresh young tomato leaves in oviposition cages for 48 h. After oviposition, egg-bearing leaves were transferred to smaller rearing cages for hatching. Newly emerged larvae were supplied daily with fresh tomato leaves. All insect cultures were maintained in a controlled-environment chamber (LRH-400A, Guangdong THK Scientific Instruments Co., Ltd., Guangzhou, China) at 25 ± 1 °C, 84 ± 1% relative humidity, and a 16:8 h (light:dark) photoperiod.

### 2.2. Conidial Preparation

The fungal strain *M. anisopliae* QLSMA201102 was maintained on potato dextrose agar (PDA) slants at 4 °C at the Resource and Utilisation Research Center of Medicinal Cordyceps, Guizhou University of Traditional Chinese Medicine. The strain was originally isolated in 2019 from naturally infected adults of Carabidae collected in Qianling Mountain Park, Guiyang City, Guizhou Province, China (106°40′21″ E, 26°59′60″ N).

For activation, the fungus was cultured on PDA plates at 25 ± 1 °C, 75% RH, and a 16:8 h (L:D) photoperiod for 10–15 days until abundant conidia were produced. Conidia were harvested by gently scraping the colony surface into 10 mL of sterile 0.02% Tween-80 solution (Beijing Solaibao Technology Co., Ltd., Beijing, China) and stored at 4 °C until use. Conidial viability was assessed by a germination assay; conidia were considered germinated when the germ tube length exceeded twice the conidial diameter. Only conidial suspensions showing ≥95% germination after 18–24 h at 25 ± 1 °C were used in subsequent experiments. Conidial concentration was determined using a Neubauer Haemocytometer (Shanghai Anxin Optical Instrument Manufacturing Co., Ltd., Shanghai, China) and adjusted to 1 × 10^8^ conidia/mL. Serial dilutions were prepared as required for each bioassay.

### 2.3. Effect of Surfactants on Conidial Germination

The compatibility of *M. anisopliae* conidia with 21 commercial surfactants was evaluated, as summarized in Table 1. The surfactants represented four major classes: non-ionic, anionic, biosurfactants, and a silicone-based type. This broad initial screening was designed to identify candidate surfactants suitable for use as formulation adjuvants to improve conidial dispersion, adhesion, and overall performance in biocontrol applications. Conidial germination was evaluated in liquid medium containing 2% sucrose, 0.5% peptone, 1% test surfactant, and 0.05% sodium carboxymethylcellulose. The medium was autoclaved at 121 °C for 20 min and cooled to room temperature before use. Conidia from a stock suspension (1 × 10^7^ conidia mL^−1^) were added to obtain a final concentration of 2 × 10^6^ conidia mL^−1^. The suspensions were incubated at 26 ± 1 °C, with shaking at 220 rpm for 24 h. A surfactant-free medium served as the control. For each replicate, more than 100 conidia were examined under a light microscope and conidia were considered germinated when the germ tube length exceeded twice the conidial diameter. Each treatment was replicated five times. The germination rate was calculated as the percentage of conidia that germinated in each replicate.

### 2.4. Effect of Surfactants on Mycelial Growth

Surfactants that did not severely inhibit conidial germination in Section 2.3 were selected for further evaluation in a mycelial growth assay. The selected surfactants were incorporated into Sabouraud Dextrose Agar supplemented with yeast extract (SDAY; per liter: 10 g yeast extract, 40 g glucose, 10 g peptone, and 15–20 g agar) at a final concentration of 1% (*w*/*v*). Surfactant-free SDAY served as controls. A 1 µL droplet of conidial suspension (1 × 10^7^ conidia mL^−1^) was placed at the center of each plate. Each replicate consisted of a single agar plate, with five replicate plates prepared per treatment. Plates were incubated at 25 ± 1 °C, and colony diameters were measured on 14 d along two perpendicular axes. The mean of the two measurements was used as the colony diameter for each replicate.

### 2.5. Effect of Surfactants on Conidia Adhesion to Tomato Leaves

Surfactants that did not significantly inhibit colony radial growth were further assessed for their effect on foliar adhesion of conidia. For each surfactant, a 1% stock solution was prepared in distilled water and brought to a final volume of 1000 mL. The solution was sterilized by autoclaving at 121 °C for 20 min, cooled to room temperature, and mixed with conidia to obtain a final concentration of 2 × 10^6^ conidia mL^−1^ from a 1 × 10^8^ conidia mL^−1^ stock suspension. Tomato plants aged 6–8 weeks and showing uniform growth were selected. From each plant, the third compound leaf from the apical growing point was excised. A total of five plants were used, with one leaf taken per plant. Each excised leaf was cleaned, immersed in the conidial-surfactant suspension for 10 s, then removed using sterile forceps, placed on sterile filter paper, and air-dried until no visible moisture remained. Five leaflets from each compound leaf were then gently pressed, adaxial side down, onto transparent adhesive tape to collect adhered conidia. The tape was mounted on a glass slide and examined under a light microscope. Conidia were counted in 10 randomly selected microscopic fields per slide. conidial suspension without surfactant served as the control.

### 2.6. Effect of Heat Stress in Aqueous Suspension on Conidial Germination

Surfactants showing poor performance in the foliar adhesion assay were excluded from further stress-tolerance evaluations. Aliquots of 1 mL conidial suspension (1 × 10^7^ conidia mL^−1^) were transferred to 2 mL screw-cap tubes and exposed to 45 ± 0.2 °C in a water bath for 45 min. This assay evaluated acute thermal tolerance of conidia in liquid suspension, simulating short-term high-temperature exposure during storage, transport or holding within spray tanks; it does not mimic complex field temperature-humidity conditions. Untreated suspensions served as controls. After heat treatment, 6 μL of each suspension was inoculated onto microscope slides coated with a thin layer of SDAY. The slides were placed in 9 cm Petri dishes lined with moistened filter paper containing 500 μL sterile distilled water and incubated in darkness at 25 °C for 24 h. Conidial germination was then assessed microscopically. Control suspensions (unheated) were treated identically. Conidia were considered germinated if its germ tube length exceeded half the conidial diameter. Each treatment was replicated five times, and 100 conidia were examined per replicate. The relative germination after heat stress was calculated for each surfactant treatment.

### 2.7. Effect of UV Radiation on Conidial Germination

Surfactants that passed the prior compatibility, adhesion, and heat-tolerance screening steps were further evaluated for their ability to reduce UV-induced damage to conidia. For each selected surfactant, 100 μL of conidial suspension (1 × 10^6^ conidia mL^−1^) was evenly spread onto microscope slides with SDAY. The prepared slides were positioned 10 cm below SolarRaptor T5 UV-A and UV-B strip-lights (Dongguan Chuangu Lighting Technology Co., Ltd., Dongguan, China). The average irradiance at a distance of 10 cm was 592.3 µW/cm^2^ for UV-B and 850 µW/cm^2^ for UV-A. After exposure for 3 h, the slides were incubated in darkness at 25 °C for 24 h. Untreated slides served as controls. Conidia were considered germinated if its germ tube length exceeded half the conidial diameter. Germination rate was determined by examining 100 randomly selected conidia per replicate, with five replicates per treatment.

### 2.8. Effect of Surfactants on Conidial Virulence

Fresh conidia were suspended in sterile aqueous solutions of each selected surfactant at final concentrations of 0.1%, 0.25%, and 0.5% (*v*/*v*). The suspensions were homogenized by vigorous shaking, filtered through 500-mesh nylon cloth, and adjusted to 1 × 10^5^ conidia mL^−1^ using a haemocytometer.

Third-instar larvae of *P. absoluta* were placed on sterile 500-mesh nylon cloth and immersed in each test suspension for 10 s. Treated larvae were blotted dry on sterile filter paper and transferred to rearing containers supplied with fresh tomato leaves and moist cotton to maintain humidity [31]. Three control treatments were included: (i) conidial suspension without surfactant, (ii) sterile water only, and (iii) surfactant solution without conidia. Each treatment consisted of three replicates of 20 larvae each. Mortality of *P. absoluta* larvae was recorded daily for 7 d. Dead larvae were surface-sterilized and placed on moist filter paper to confirm mycosis by outgrowth of *M. anisopliae*. Because the primary comparison was between fungus + surfactant and fungus-alone treatments, cumulative mortality was used in this study.

### 2.9. Statistics and Analysis

Data was compiled in Microsoft Excel 2023 and analyzed in R version 4.4.1 [32]. Results are presented as mean ± standard deviation (SD). Proportion-derived endpoints (conidial germination under surfactants and larval mortality) were analyzed using the original counts of successes and failures via generalized linear models (GLMs) with a binomial error distribution and logit link function. For the 7-day cumulative mortality, a quasibinomial GLM was used to account for residual non-uniformity. For germination under heat and UV stress, binomial generalized linear mixed models (GLMMs) with an observation-level random effect were applied to account for overdispersion. Continuous endpoints (colony diameter, foliar adherence, and dispersibility) were analyzed using Gaussian GLMs. Model fit was assessed with the DHARMa package. Estimated marginal means (EMMs) were obtained using the emmeans package. Treatment-to-control comparisons were performed using EMMs with Bonferroni adjustment for germination, colony diameter, foliar adherence, dispersibility, and stress tolerance assays. For cumulative larval mortality, Dunnett adjustment was used because all surfactant–fungus treatments were compared with a single fungus-alone control.

## 3. Results

### 3.1. Effect of Surfactants on Conidial Germination and Radial Growth

**Figure 1:**
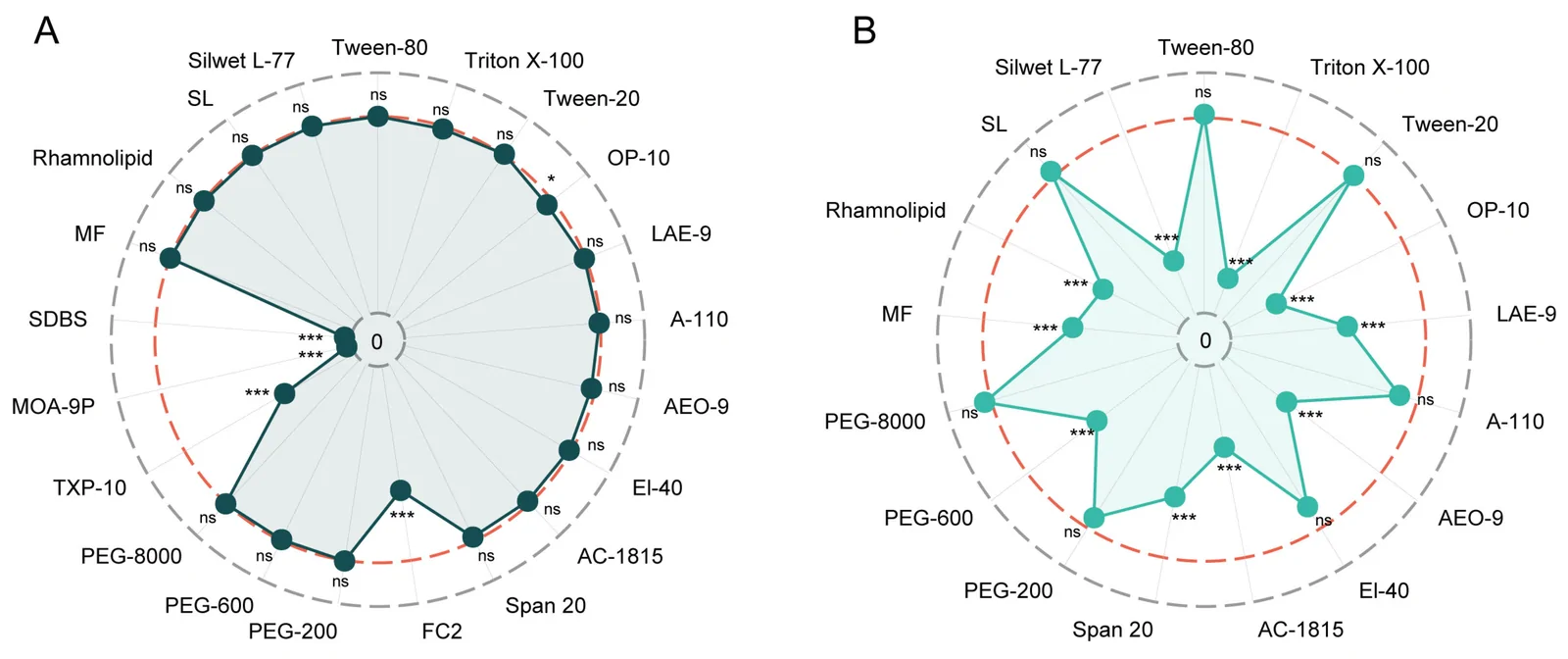
Effect of different surfactants on *M. anisopliae* QLSMA201102 conidia. (**A**) Germination rate of conidia following exposure to 21 surfactants. (**B**) Radial colony growth on SDAY medium. Values represent mean ± SD (*n* = 5). For germination (**A**), data were analyzed using a binomial GLM with logit link, and treatment-to-control comparisons were performed using estimated marginal means with Bonferroni adjustment. The red dotted line indicates the control mean. Asterisks indicate significant differences compared with the control: * *p* < 0.05, *** *p* < 0.001; ns, not significant.

Conidial viability and radial growth are critical indicators of fungal performance in biocontrol applications. The effect of 21 commercial surfactants on the germination of *M. anisopliae* QLSMA201102 conidia is presented in Figure 1A. Most non-ionic surfactants, biosurfactants, and the silicone surfactant Silwet L-77 did not significantly affect conidial germination compared to the control (Bonferroni-adjusted *p* > 0.05). In contrast, several anionic surfactants strongly inhibited germination: MOA-9P, SDBS, and TXP-10 reduced germination to 2.62%, 3.42%, and 40.51%, respectively, all significantly lower than the control (Bonferroni-adjusted *p* < 0.0001). Within the non-ionic class, FC2 and OP-10 also significantly reduced germination, with predicted rates of 62.81% and 94.76%, respectively (Bonferroni-adjusted *p* < 0.0001 for FC2 and *p* = 0.0230 for OP-10).

Radial growth assays on SDAY medium revealed further compatibility effects (Figure 1B). Among non-ionic surfactants, Triton X-100, OP-10, AEO-9, AC-1815, LAE-9, PEG-600, and Span 20 significantly reduced colony diameter compared with the control (Bonferroni-adjusted *p* < 0.05), whereas Tween-80, Tween-20, PEG-200, PEG-8000, A-110, and El-40 did not differ significantly from the control (Bonferroni-adjusted *p* > 0.05). PEG-8000 showed a numerically larger colony diameter than the control, but the difference was not statistically significant after Bonferroni correction. Within the anionic surfactants, MF significantly inhibited growth (Bonferroni-adjusted *p* < 0.0001). For biosurfactants, Rhamnolipid significantly reduced colony diameter (Bonferroni-adjusted *p* < 0.0001), whereas SL did not differ significantly from the control (Bonferroni-adjusted *p* > 0.05). The silicone-based surfactant Silwet L-77 also strongly suppressed radial growth (*p* < 0.0001).

### 3.2. Effect of Surfactants on Conidial Foliar Adherence and Dispersibility

**Figure 2:**
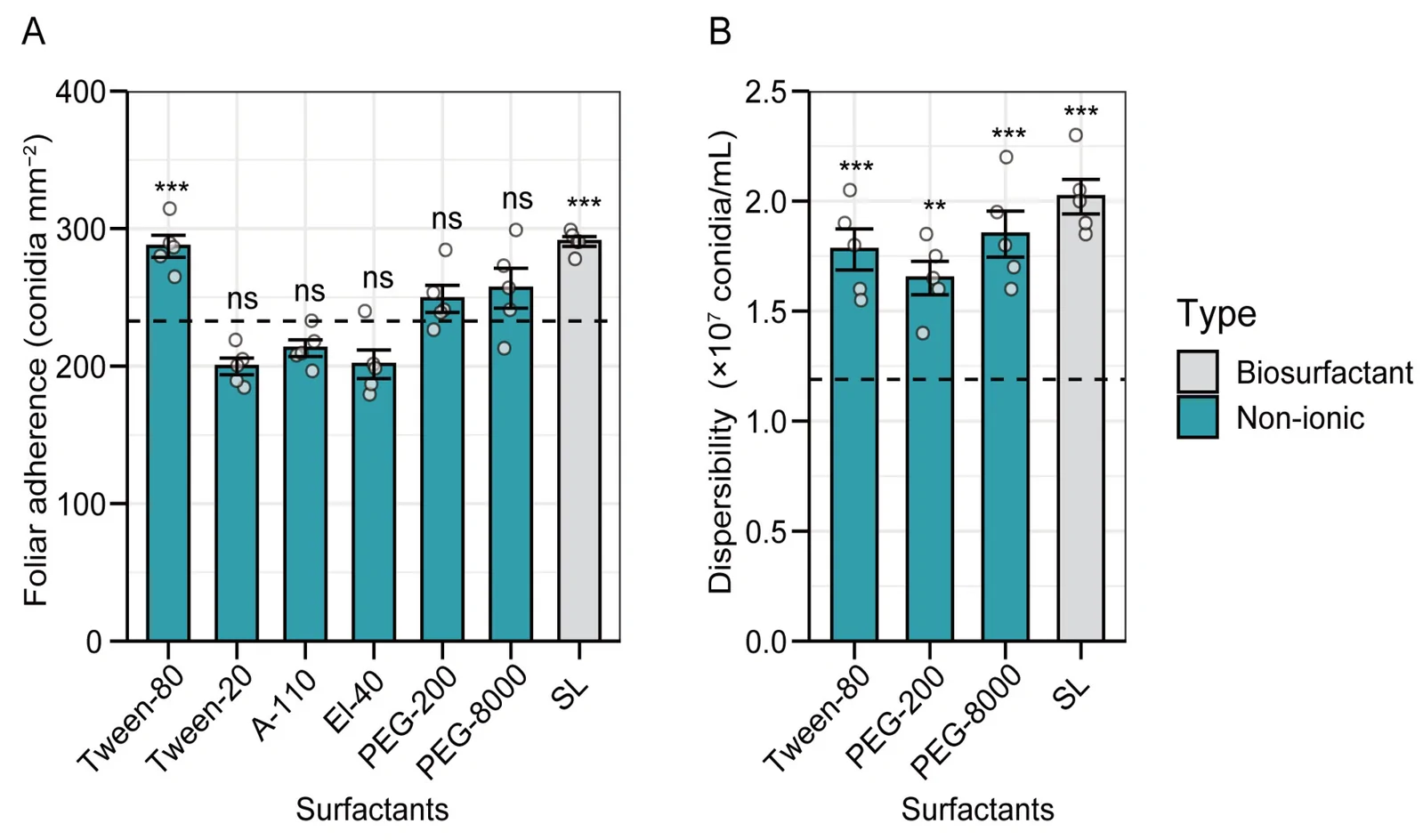
Effect of selected surfactants on conidial retention on tomato leaves and dispersibility in aqueous suspension. (**A**) Foliar adherence of *M. anisopliae* QLSMA201102 conidia, expressed as the number of conidia adhered per mm^2^ following immersion of tomato leaves in conidial suspensions (2 × 10^6^ conidia mL^−1^) containing 1% surfactant. (**B**) Dispersibility of conidia in aqueous suspension (1 × 10^7^ conidia mL^−1^) after homogenization. Values represent mean ± SD (*n* = 5). The dashed horizontal line indicates the mean value of the control treatment. Data were analyzed using Gaussian GLMs, and treatment-to-control comparisons were performed using estimated marginal means with Bonferroni adjustment. Asterisks indicate significant differences compared with the control: ** *p* < 0.01, *** *p* < 0.001; ns, not significant.

Surfactants that showed acceptable compatibility in the viability and growth assays were further evaluated for their ability to improve conidial retention on tomato leaves and dispersibility in aqueous suspension. Conidial foliar adherence varied significantly among surfactant treatments (Figure 2A). Compared with the control (232.8 conidia mm^−2^), the biosurfactant SL and the non-ionic surfactant Tween-80 significantly increased conidial retention on tomato leaves, reaching 290.6 and 287.1 conidia mm^−2^, respectively (Bonferroni-adjusted *p* = 0.0008 for SL and *p* = 0.0018 for Tween-80). In contrast, A-110, El-40, PEG-200, PEG-8000, and Tween-20 did not differ significantly from the control after Bonferroni correction (*p* > 0.05), although moderate numerical variation among treatments was observed.

Surfactant treatments also significantly influenced the dispersibility of conidia in suspension (Figure 2B). All tested surfactants improved dispersibility relative to the control, with statistically significant increases observed for PEG-200 (*p* = 0.0046), PEG-8000 (*p* = 0.0001), SL (*p* < 0.0001), and Tween-80 (*p* = 0.0004). Among them, SL exhibited the highest dispersibility, followed by PEG-8000, Tween-80, and PEG-200. SL and Tween-80 improved both foliar adherence and dispersibility. Based on their favorable performance in both foliar adherence and dispersibility assays, selected surfactants were further evaluated for their effects on conidial tolerance to environmental stresses and insecticidal efficacy.

### 3.3. Effect of Surfactants on Conidial Tolerance to Heat and UV Radiation

**Figure 3:**
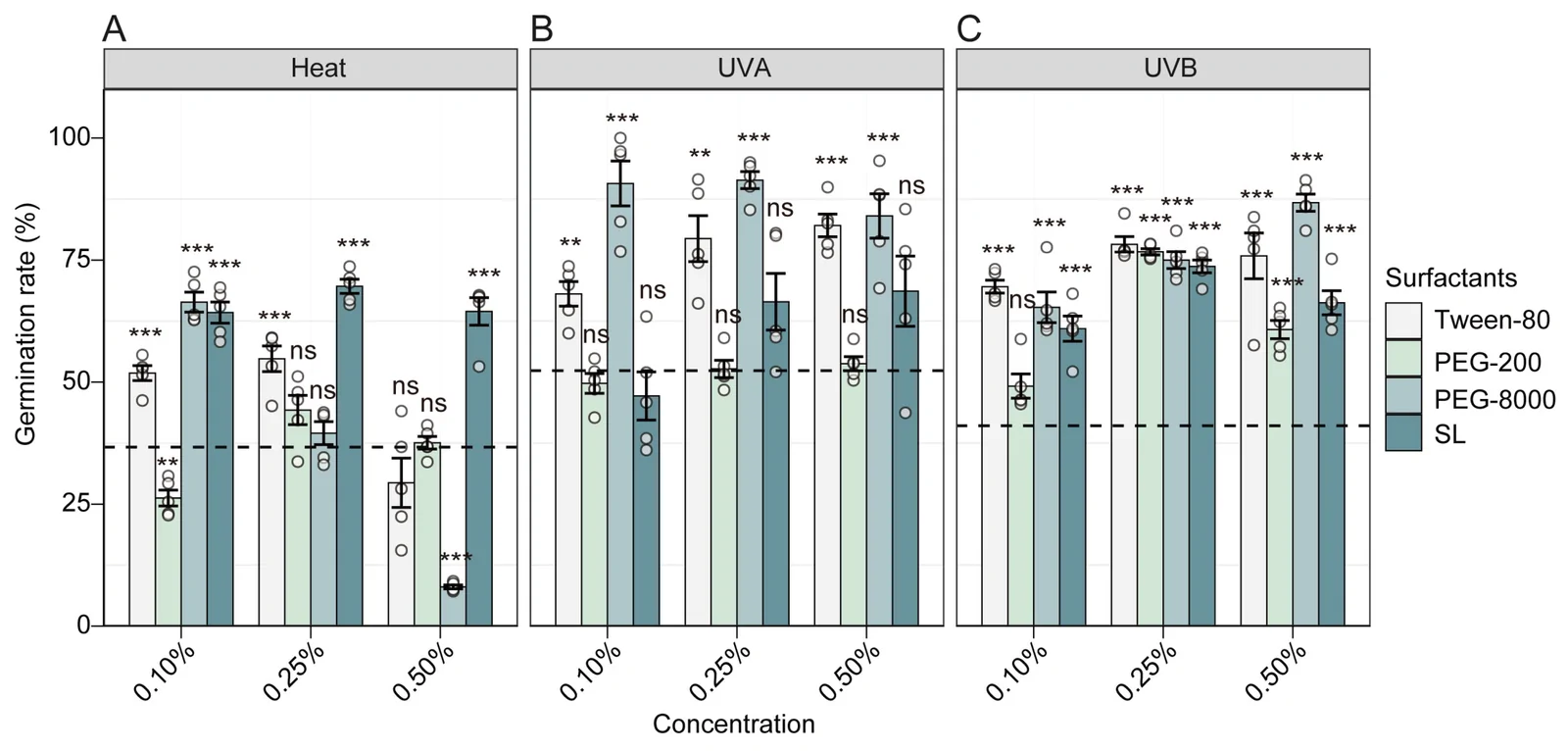
Effect of selected surfactants at different concentrations on heat and ultraviolet tolerance of *M. anisopliae* QLSMA201102 conidia. (**A**) Germination after exposure to heat stress. (**B**) Germination following UV-A irradiation. (**C**) Germination following UV-B irradiation. Values represent mean ± SD (*n* = 5). The dashed horizontal line indicates the mean value of the control treatment. Data were analyzed using binomial GLMMs with an observation-level random effect to account for overdispersion; treatment-to-control comparisons used estimated marginal means with Bonferroni adjustment. ** *p* < 0.01, *** *p* < 0.001; ns, not significant.

The four surfactants that performed best in previous assays (PEG-200, Tween-80, PEG-8000, and SL) were further evaluated for their effects on conidial tolerance to thermal and ultraviolet stresses (Figure 3). Under heat stress, surfactant treatments significantly affected conidial germination (Figure 3A). At 0.1%, PEG-8000, SL, and Tween-80 significantly increased germination compared with the control (Bonferroni-adjusted *p* < 0.001 for PEG-8000 and SL, *p* = 0.0002 for Tween-80), whereas PEG-200 significantly reduced germination (Bonferroni-adjusted *p* = 0.0097). At 0.25%, SL and Tween-80 significantly increased germination (Bonferroni-adjusted *p* < 0.0001 for both), whereas PEG-200 and PEG-8000 did not differ significantly from the control (*p* > 0.05). At 0.5%, SL significantly increased germination (Bonferroni-adjusted *p* < 0.0001), whereas PEG-8000 significantly suppressed germination (Bonferroni-adjusted *p* < 0.0001) and Tween-80 did not differ significantly from the control (Bonferroni-adjusted *p* > 0.05). Overall, the highest germination rate under heat stress (69.6%) was obtained with 0.25% SL, which was substantially higher than the control (36.7%).

Under UV-A exposure (Figure 3B), surfactant treatments also influenced conidial survival. PEG-8000 consistently exhibited the strongest protective effect, with predicted germination rates of 90.7%, 91.4%, and 84.2% at 0.10%, 0.25%, and 0.50%, respectively (Bonferroni-adjusted *p* < 0.0001, *p* = 0.0221, and *p* = 0.0257). Tween-80 significantly improved germination at 0.25% and 0.50% (Bonferroni-adjusted *p* = 0.0161 and *p* = 0.0059), but not at 0.10%. In contrast, neither PEG-200 nor SL differed significantly from the control at any tested concentration (Bonferroni-adjusted *p* > 0.05). Overall, the relative effectiveness of surfactants against UV-A stress followed the general trend: PEG-8000 > Tween-80 > SL ≈ PEG-200.

Exposure to UV-B radiation produced similar but slightly stronger protective responses (Figure 3C). At 0.1%, Tween-80, PEG-8000, and SL significantly increased germination compared with the control (Bonferroni-adjusted *p* < 0.0001), whereas PEG-200 did not differ significantly from the control (Bonferroni-adjusted *p* = 0.6749). At 0.25%, all four surfactants significantly enhanced germination relative to the control (Bonferroni-adjusted *p* < 0.0001). A similar pattern was observed at 0.5%, with all treatments providing significant protection against UV-B stress (Bonferroni-adjusted *p* < 0.0001). Among the tested surfactants, PEG-8000 showed the clearest concentration-dependent response, with predicted germination increasing from 65.3% at 0.1% to 86.8% at 0.5%. In contrast, the other surfactants displayed less consistent concentration responses. Based on their favorable performance in dispersibility, foliar adherence, and stress-tolerance assays, selected surfactants were further evaluated for their influence on the insecticidal efficacy of *M. anisopliae* against *P. absoluta* larvae.

### 3.4. Effects of Surfactants on Conidial Virulence Against P. absoluta

**Figure 4:**
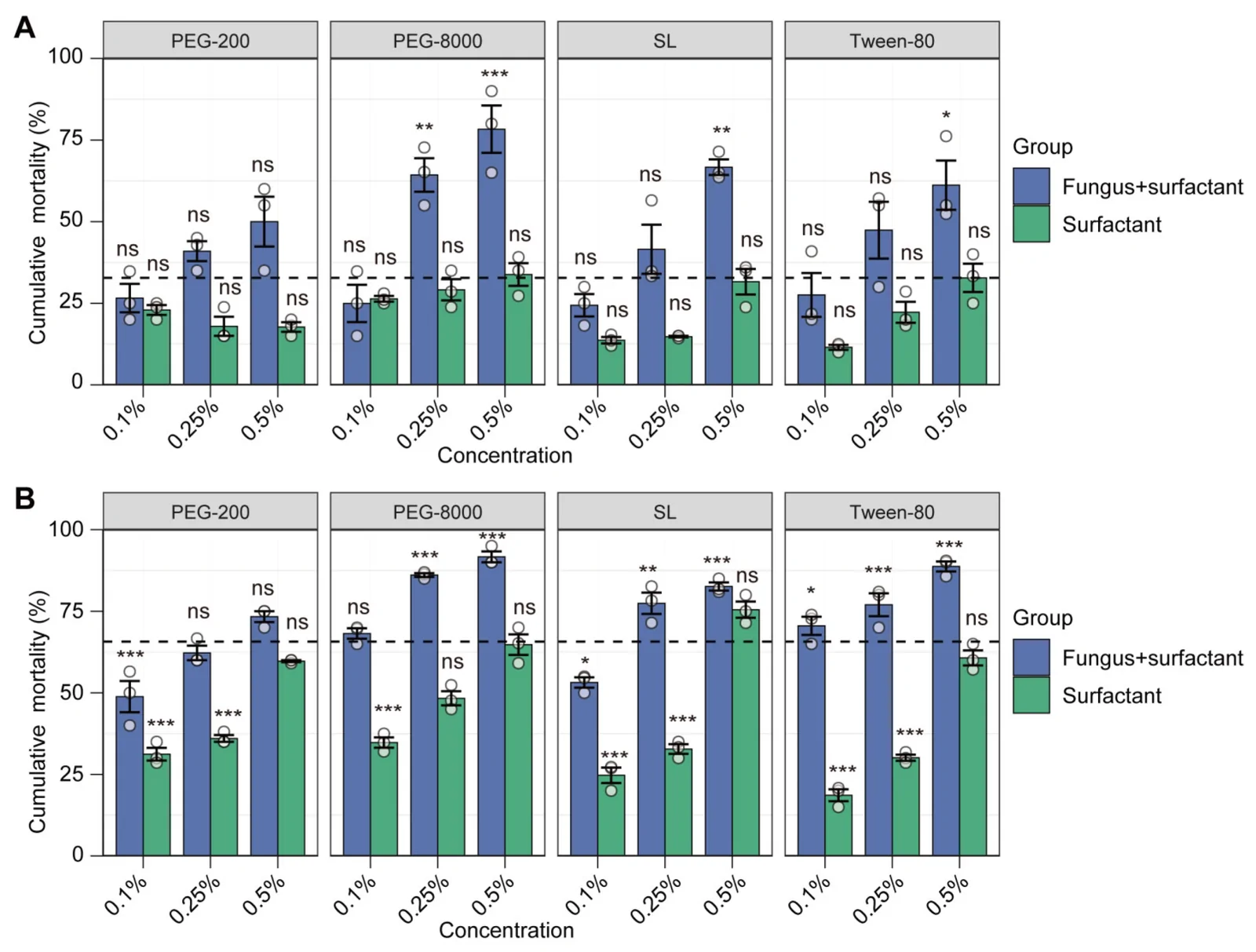
Effects of selected surfactants on the virulence of *M. anisopliae* QLSMA201102 against *P. absoluta* larvae. (**A**) Cumulative mortality of third-instar larvae 3 days after immersion in conidial suspensions (1 × 10^5^ conidia mL^−1^) containing 0.1%, 0.25%, or 0.5% surfactant. (**B**) Cumulative mortality of third-instar larvae 7 days after treatment with the same suspensions. Values represent mean ± SD (*n* = 3). The dashed horizontal line indicates the mean mortality of the conidia-only control. Mortality data were analyzed using binomial GLMs with logit link (3 days) and quasibinomial GLM with logit link (7 days); treatment-to-control comparisons used estimated marginal means with Dunnett adjustment. * *p* < 0.05, ** *p* < 0.01, *** *p* < 0.001; ns, not significant.

Bioassays with third-instar *P. absoluta* larvae treated with *M. anisopliae* conidial suspensions supplemented with surfactants (0.1–0.5%) revealed significant differences in insect mortality among treatments (Figure 4). Based on the 3-day data (Figure 4A), several surfactant-fungus combinations enhanced early insecticidal activity relative to the fungus-alone control. At the highest concentration (0.5%), SL + fungus, PEG-8000 + fungus, and Tween-80 + fungus significantly increased mortality. In contrast, PEG-200 + fungus produced only a moderate, non-significant increase in mortality (*p* = 0.4591). Among the tested treatments, PEG-8000 + fungus also significantly improved early mortality at 0.25% (*p* = 0.0094).

Analysis of the 7-day cumulative mortality data (Figure 4B) revealed stronger treatment effects over time. Compared with the fungus-alone control, fungus + PEG-8000 at 0.25% and 0.5% produced the strongest and most consistent increases in larval mortality (*p* < 0.0001 for both). Fungus + Tween-80 at 0.5% also significantly increased mortality (*p* < 0.0001), followed by fungus + SL at 0.5% (*p* < 0.0001), fungus + SL at 0.25% (*p* = 0.0093), and fungus + Tween-80 at 0.25% (*p* = 0.0223). In contrast, PEG-200 + fungus did not significantly increase mortality at any concentration (*p* > 0.05), and fungus + SL at 0.1% significantly reduced mortality relative to the fungus-alone control (*p* = 0.0230). At the lowest concentration (0.1%), none of the other surfactant-fungus combinations produced statistically significant improvements in mortality (Dunnett-adjusted *p* > 0.05).

Across all assays, SL, PEG-8000, and Tween-80 consistently demonstrated high compatibility with *M. anisopliae* conidia and improved key formulation traits, including foliar adherence, dispersibility, environmental stress tolerance, and insecticidal efficacy. In contrast, PEG-200 showed limited benefits across multiple performance indicators.

## 4. Discussion

The most promising formulation additives were PEG-8000, Tween-80, and the biosurfactant SL, which maintained high conidial germination and improved dispersibility and foliar adherence. In contrast, several anionic surfactants strongly inhibited germination. SDBS, MOA-9P, and TXP-10 severely inhibited conidial germination, whereas MF exhibited relatively neutral effects on conidial viability. This pattern likely reflects differences in alkyl chain length and membrane-interacting properties among these surfactants, as long-chain surfactants can penetrate lipid bilayers and disrupt fungal cell membranes. Previous work has shown that surfactants with extended hydrophobic chains can increase membrane permeability, leading to cytoplasmic leakage and reduced fungal viability [33]. These findings highlight that surfactant classification alone is insufficient to predict compatibility, and molecular structure must be considered when selecting formulation adjuvants for fungal biopesticides. Previous studies have demonstrated that the compatibility of anionic surfactants with entomopathogenic fungi is highly dependent on their molecular structure and concentration. For example, Melo et al. reported that the anionic surfactant Morwet 3020 completely inhibited the growth and germination of *Beauveria bassiana*, indicating strong cytotoxic effects [16]. Conversely, other anionic surfactants can be compatible at low concentrations, highlighting the importance of molecular architecture in determining biological effects [16].

Conidial germination and mycelial growth are fundamental indicators of fungal viability and are widely used to evaluate compatibility between formulation additives and entomopathogenic fungi [34]. Our screening approach demonstrated that several surfactants that did not strongly inhibit germination still significantly reduced mycelial growth, emphasizing the importance of multi-parameter compatibility assessment. Interestingly, the silicone-based surfactant Silwet L-77 significantly reduced the viability of *M. anisopliae* QLSMA201102, despite previous reports indicating high compatibility with *B. bassiana* and *Isaria fumosorosea* [35]. This inhibitory effect may stem from strain-specific sensitivity of fungal cell membranes toward organosilicone compounds. Polyether-modified polydimethylsiloxane (Silwet L-77) can interact with membrane lipids, elevate membrane permeability, and trigger cytoplasmic leakage in fungal cells. Moreover, our initial compatibility screening adopted a relatively high 1% surfactant concentration, which may exceed the tolerance threshold of this specific strain, while many published compatible results for other fungal strains were obtained under lower concentrations. This observation stresses that compatibility outcomes cannot be generalized across strains; both strain-specific testing and concentration-gradient evaluation are indispensable for adjuvant screening.

In addition to compatibility, effective formulation must enhance the dispersibility and deposition of conidia on plant surfaces, as foliar spray application is a common delivery route for fungal biocontrol agents in agriculture. Surfactants reduce surface tension and improve the wettability and dispersion of hydrophobic conidia, thereby enhancing spray coverage and adherence to plant surfaces, which may improve the probability of conidial contact with target pests [15,16,36]. In the present study, Tween 80, PEG-8000, PEG-200 and the biosurfactant SL significantly improved conidial dispersibility and foliar retention, which are key determinants of infection efficiency in field conditions. These findings are consistent with previous work demonstrating that non-ionic surfactants such as Tween-80 can enhance conidial suspension quality and improve deposition on plant surfaces [32]. However, species-specific responses were also observed, as rhamnolipid inhibited the bioactivity of *M. anisopliae* despite reports of positive effects on *B. bassiana* [37]. These contrasting responses further emphasize that surfactant compatibility must be empirically evaluated for each fungal biocontrol system.

Environmental stresses such as elevated temperatures and ultraviolet radiation are major constraints on the field persistence and efficacy of fungal biocontrol agents [38,39,40]. High temperatures can impair conidial germination, mycelial growth, and overall infectivity, and thermal optima for major entomopathogenic fungi generally lie around 25–30 °C, with performance declining under heat stress [40,41,42]. Additionally, ultraviolet radiation can rapidly inactivate conidia on exposed plant surfaces; under UV-B exposure, viability may decline markedly within several hours, thereby reducing field persistence and efficacy [38,39,43]. Our results demonstrate that certain surfactants can partially mitigate these environmental stresses, with PEG-8000 providing strong protection against UV radiation and the biosurfactant SL significantly improving thermotolerance. It should be noted that our aqueous-suspension heat-stress assay using 45 °C water-bath incubation represents only a laboratory screening tool. This setup simulates short-term high-temperature exposure that may occur during product storage, transportation, or holding within spray tanks, rather than complex combined temperature-humidity conditions under real-world field environments. These protective effects were concentration-dependent, suggesting that optimal surfactant dosage is critical for balancing formulation performance and fungal viability.

Finally, surfactants influenced not only the physicochemical behavior of the formulation but also the biological performance of *M. anisopliae* against *P. absoluta*. Recent EPF formulation studies show that adjuvants must improve wetting, dispersion, and delivery of hydrophobic conidia without compromising fungal viability or virulence, and that these effects are strongly dependent on surfactant chemistry and concentration [16,20]. Consistent with this interpretation, Zeina and Laing found that some non-ionic surfactants, including Tween 80 and Silwet L-77, maintained or enhanced conidial viability at low concentrations, whereas other adjuvants inhibited germination or failed to improve pathogenicity; importantly, only a subset improved host mortality, showing that physicochemical compatibility does not necessarily translate into enhanced biological efficacy [25]. Comparable interactions have been reported in other pest systems, where surfactant-assisted formulations supported effective conidial dispersion and high mortality against *Bemisia tabaci*, and where Silwet L-77 combined with EPF increased mortality, reduced LT_50_ and increased mycosis in *Spodoptera frugiperda* [44,45]. Consistent with these studies, our results showed that SL, PEG-8000, and Tween-80 enhanced the virulence of *M. anisopliae* against *P. absoluta* larvae, but the magnitude of enhancement depended on concentration. At 0.1%, which is closer to practical field rates, Tween-80 produced the largest increase in mortality, followed by SL, whereas PEG-8000 showed only modest improvement. In contrast, the higher 0.5% concentration also caused direct surfactant mortality, suggesting that excessive adjuvant loading can confound the apparent fungal effect. Because EPF infection requires sufficient conidial attachment and penetration of the host cuticle, the improved efficacy observed here likely reflects better conidial spreading and greater opportunity for contact with the larval integument, although some contribution of direct surfactant toxicity at higher concentrations cannot be excluded [20,21]. Overall, these findings show that adjuvant selection for *M. anisopliae* formulations should be based not only on compatibility, but also on concentration-specific bioefficacy, so that improved delivery is achieved without sacrificing fungal performance or inflating direct surfactant toxicity. However, this study has several limitations: formulation performance was assessed under laboratory conditions only; larval immersion, although ensuring uniform conidial exposure, does not mimic foliar spray application in the field; and the virulence assay had only three independent biological replicates, with additional repetitions constrained by limited availability of synchronized third-instar larvae, the large number of surfactant-treatment combinations, and time and funding constraints. Therefore, further greenhouse and field experiments using spray application are needed to confirm the persistence, efficacy, and practical applicability of the selected surfactant-conidia combinations under commercial tomato production systems.

## 5. Conclusions

This study demonstrates that surfactant selection is a critical determinant of formulation performance in entomopathogenic fungal biocontrol systems. Among the 21 commercial surfactants spanning four chemical classes screened in this study, the non-ionic surfactants PEG-8000 and Tween-80, together with the biosurfactant sophorolipid (SL), exhibited high biological compatibility with *M. anisopliae* strain QLSMA201102. These additives preserved high conidial germination capacity while improving multiple formulation-relevant performance metrics.

PEG-8000 delivered prominent protective effects against UV-A and UV-B radiation; sophorolipid (SL) markedly enhanced conidial thermotolerance under aqueous heat-stress conditions. Both surfactants, alongside Tween-80, significantly improved conidial dispersibility and foliar adherence on tomato leaf surfaces.

Supplementing *M. anisopliae* conidia with PEG-8000, Tween-80 or SL at 0.5% concentration significantly enhanced larval mortality against *P. absoluta* in laboratory immersion bioassays, achieving cumulative mortality values above 91% at 7 days post-treatment.

Several anionic surfactants showed strong inhibitory activity toward conidial germination, demonstrating that surfactant chemical classification alone cannot predict biological compatibility with entomopathogenic fungi; empirical strain-specific screening is indispensable for adjuvant selection.

This laboratory-based investigation provides baseline evidence for surfactant selection for *M. anisopliae* formulation. Further greenhouse and open-field trials are required to validate formulation stability, stress resilience and pest control efficacy under practical tomato production conditions.

## Figures and Tables

**Table 1:** Physicochemical characteristics of the surfactants evaluated in this study.

Surfactant	Type	Chemical Composition	Manufacturer	HLB
Tween-80	Non-ionic	Sorbitan monooleate polyoxyethylene (80)	BS	15.6
Triton X-100		Octylphenol ethoxylate	BS	13.4
Tween-20		Polyoxyethylene (20) sorbitan monolaurate	BS	16.7
OP-10		Octylphenol ethoxylate	HP	13.3–14
LAE-9		Polyoxyethylene fatty acid ester	HP	13–14
PEG-600		Polyethylene glycol 600	HP	10–11
A-110		Fatty acid polyoxyethylene ester	HP	12–13
AEO-9		Fatty alcohol polyoxyethylene ether	HP	13–14
E1-40		Polyoxyethylene castor oil	HP	13–14
AC-1815		Fatty amine polyoxyethylene ether	HP	14–15
Span-20		Sorbitan monolaurate	AS	8.6
FC-2		Poly(oxy-1,2-ethanediyl), α-isodecyl-ω-hydroxy	AS	11.5
PEG-8000		Polyethylene glycol 8000	BS	NA
PEG-200		Polyethylene glycol 200	BS	9.5
TXP-10	Anionic	Phenol ether phosphate ester	HP	NA
MOA-9P		Fatty alcohol polyoxyethylene (9) ether	HP	NA
SDBS		Sodium dodecylbenzene sulfonate	SM	10.6
MF		Sodium methyldimethyl naphthalene sulfonate	SM	NA
Rhamnolipid R90	Biosurfactants	Rhamnolipid biosurfactant	SM	10–15
SL		Sophorolipid (1′,4″-sophorolactone-6′,6″-diacetate)	SM	9–12
Silwet L-77	Silicone	Polyether-modified polydimethylsiloxane	BS	5–8

BS: Beijing Solarbio Science & Technology Co., Ltd., Beijing, China. HP: Hai’an Petrochemical Plant, Nantong, Jiangsu, China. AS: Aladdin Biochemical Technology Co., Ltd., Shanghai, China. SM: Shanghai Macklin Biochemical Co., Ltd., Shanghai, China. HLB: Hydrophilic–lipophilic balance. NA: Not available from manufacturer specifications.

## Data Availability

The original contributions presented in the study are included in the article; further inquiries can be directed to the corresponding authors.
